# Subclinical Diastolic Dysfunction Precedes Decline in Renal Function: Cause, Effect, or Shared Etiology?

**DOI:** 10.34067/KID.0000000000000112

**Published:** 2023-05-25

**Authors:** Jacob Christensen

**Affiliations:** Center for Translational Cardiology and Pragmatic Randomized Trials (CTCPR), Department of Cardiology, Copenhagen University Hospital Gentofte, Hellerup, Denmark

**Keywords:** cardiovascular

Cardiovascular and renal physiology are closely linked, and disruptions in one system can have significant effect on the functioning of the other. Accordingly, advanced disease states in these systems, such as congestive heart failure and CKD, likely precipitate and exacerbate one another.^1^ Although the subject is a highly active field of research, efforts to establish the roles of involved processes are complicated by the reciprocal and complex nature of the cardiorenal relationship. Consequently, much remains to be elucidated regarding the concomitant development and progression of cardiac and renal dysfunction.

In this issue, Mehta *et al.* present their work with the Cardiovascular Health Study,^2^ in which they investigated associations between echocardiographic parameters and eGFR in an older population 65 years or older and free of known HF. The study design included both cross-sectional (*N* of subjects=3516) and longitudinal analysis (*n* of subjects=2135), which examined baseline echocardiographic parameters in relation to the presence of CKD at baseline and a 30% decline in eGFR over a 7-year follow-up period, respectively. In addition, sensitivity analysis was conducted to examine the association between 2D speckle-tracking echocardiography parameters and rapid decline in eGFR, defined as an annual decline rate of >3 ml/min per 1.73 m^2^ per year. Though no correlation was found between diastolic indices and decline in eGFR as continuous variables in adjusted linear regression, the study found that diastolic function indices were associated with both rapid annual decline and >30% total decline in eGFR. The authors have thus demonstrated a link between decline in renal function and subnormal diastolic function assessed by multiple echocardiographic parameters in an older general population.

The authors have conducted a scientifically sound and thorough exploratory study with strengths that include a large study population and a long follow-up period. The population-based, multicentre, and longitudinal study design of the Cardiovascular Health Study provides a robust basis for testing the hypothesis that subclinical cardiac dysfunction assessed by echocardiography is associated with decline in kidney function over time. The limitations of this study were addressed by the authors in a well-balanced discussion, in which it was highlighted that causality cannot be established from the results, and further that the findings might not be directly reproducible in contemporary settings because of medical and technological advances after the time of original data collection.

The finding from Mehta *et al.* has potential implications of both physiological and clinical significance. Physiologically, it supports a potential role of diastolic function in relation to a decrease in renal function which cannot be attributed solely to age-related decline. As formerly stated, causality cannot be established from this finding, but the extent to which it contributes to current understanding may be explored through three criteria for a causal link between two variables as proposed by Mill^3^: (*1*) temporal precedence of the independent variable, (*2*) covariance between the variables, and (*3*) exclusion of tertiary confounding factors. First, the results demonstrate temporal precedence of subclinical diastolic dysfunction before decline in eGFR. This is in line with the recent results of the German population-based Gutenberg Health Study, in which the diastolic parameter E/e was strongly associated with decline in eGFR over a 5-year period and further predicted higher rate of decline.^4^ Second, the results demonstrate covariance between diastolic function indices and prevalence of CKD at baseline. The interpretation of this, however, particularly regarding directionality of the interaction, is challenging. For instance, the Shanghai Heart Health Study found no association between clinical diastolic dysfunction and eGFR in a general older population,^5^ which would otherwise have been expected if diastolic dysfunction was able to affect kidney function. Beyond the general population, although it has previously been established that in patients with manifest CKD, diastolic dysfunction is highly prevalent and a strong predictor of mortality.^6^ In addition, diastolic function has been found to correlate with progressive stages of CKD, even in early stages,^7^ and further to gradually deteriorate in parallel with kidney function.^8^ The covariance between diastolic function and renal function thus seems strongest in subjects with manifest kidney disease. The work of Mehta *et al.* provides interesting new insight into this because it shows that baseline diastolic parameters are associated with eGFR in a general population, not only cross-sectionally but also with a longitudinal decline in eGFR. However, it seems unlikely that diastolic dysfunction is independent of renal function. Finally, although this study includes thorough adjusted analysis, the interwoven and complex nature of the cardiorenal relationship precludes the possibility of excluding all confounding factors. In continuation of this, it would, for instance, be of interest to investigate the concomitant decline of both cardiac and renal parameters because diastolic parameters might affect systolic parameters over time, which in turn might impose confounding effects on the perceived link between diastolic and renal function. In addition to the three aforementioned criteria may be added that one or several plausible underlying mechanisms for physiological interaction should be present. The authors propose several potential mechanisms to explain their results, including both direct interaction and underlying systemic mechanisms that result in adverse effects in both organ systems. The abundance of potential mechanisms highlights the need for further investigation, particularly with the aim of disproving some of the existing theories. As a final criterion for causality may be added that the relationship should involve proportional variation between diastolic and renal function, analogous to a dose-response relationship. Such proportional variation was explored in this study, and adjusted linear regression failed to show correlation between diastolic parameters and decline in eGFR as continuous variables. Although it is uncertain whether the link might instead follow a nonlinear relationship, the finding does not support causality between diastolic dysfunction and decline in renal function.

Of clinical significance, the finding by Mehta *et al.* suggests potential utility of echocardiographic assessment of diastolic function in the effort to identify at-risk subjects free of manifest CKD in whom early monitoring and treatment might be beneficial. Future studies might aim to establish a threshold for diastolic parameters at which an association with future decline in eGFR arises and which could guide the need for potential intervention. As stated by the authors, Sodium-glucose co-transporter 2 (SGLT2) inhibitors have gained widespread use, especially because of their renoprotective properties in patients with CKD and HF^9,10^ as well as cardioprotective properties.^11^ Accordingly, the latest clinical practice guidelines for the management of HF now recommend SGLT2 inhibitors for patients with HF with preserved ejection fraction (HFpEF).^12^ Because HFpEF is generally regarded a clinical manifestation of diastolic dysfunction,^13^ this leaves a narrow potential target population with subclinical diastolic dysfunction, in which intervention with SGLT2 inhibitors might be initiated and tested. Furthermore, the effects of SGLT2 inhibitors on cardiac remodeling and diastolic function still require further investigation, and the underlying processes and effects have yet to be fully established.^14^ As such, the basis for future interventional studies needs to be expanded.

In conclusion, the work by Mehta *et al.* adds to current knowledge of the cardiorenal link and contributes to laying the groundwork for future mechanistic and, perhaps in time, interventional studies. From the work can be gathered a new hypothesis that subclinical diastolic dysfunction might precipitate decline in renal function, which can now be tested and explored.
